# Do deposit-feeders compete? Isotopic niche analysis of an invasion in a species-poor system

**DOI:** 10.1038/srep09715

**Published:** 2015-05-19

**Authors:** Agnes M. L. Karlson, Elena Gorokhova, Ragnar Elmgren

**Affiliations:** 1 Dept. Environmental Science and Analytical Chemistry, Stockholm University, SE-10691 Stockholm, Sweden; 2 Dept. Ecology, Environment and Plant Sciences, Stockholm University, SE-10691 Stockholm, Sweden

## Abstract

Successful establishment of invasive species is often related to the existence of vacant niches. Competition occurs when invaders use the same limiting resources as members of the recipient community, which will be reflected in some overlap of their trophic niches. The concept of isotopic niche has been used to study trophic niche partitioning among species. Here, we present a two-year field study comparing isotopic niches of the deposit-feeding community in a naturally species-poor system. The isotopic niche analyses showed no overlap between a recent polychaete invader and any of the native species suggesting that it has occupied a vacant niche. Its narrow isotopic niche suggests specialized feeding, however, the high δ^15^N values compared to natives are most likely due to isotope fractionation effects related to nitrogen recycling and a mismatch between biological stoichiometry of the polychaete and the sediment nitrogen content. Notably, highly overlapping isotopic niches were inferred for the native species, which is surprising in a food-limited system. Therefore, our results demonstrate that invaders may broaden the community trophic diversity and enhance resource utilization, but also raise questions about the congruence between trophic and isotopic niche concepts and call for careful examination of assumptions underlying isotopic niche interpretation.

Classic theory suggests that no two species can have the same niche; the less effective competitor will be excluded from an area^1^. The basis for coexistence is niche differentiation, often obtained through resource partitioning^2^. Invasive species are often generalists^3^ that can take advantage of formerly unexploited resources, thereby occupying a distinct niche space relative to the native community. Hence, invasion success is often explained by occupation of a previously vacant niche^3^,^4^. Alternatively, the invader might share trophic niche space with some members of the native community but be competitively superior, for example, by having higher feeding rate or food conversion efficiency and hence higher production^3^,^5^. Finally, if food resources are not limiting, co-existence may result from interference competition for space^6^. The ecological strategies of invasive species can cause food web changes and influence ecosystem functioning^7^,^8^,^9^. However, there also examples of invasive species replacing natives with similar trophic niches, which results in little change in overall ecosystem functioning^10^.

Stable isotopes have become powerful tools for studying food webs and trophic relationships as they provide time-integrated information on diet and habitat use^11^. For nitrogen, ratios of ^15^N to ^14^N (expressed as δ^15^N) exhibit stepwise enrichment with trophic transfer, while ratios of carbon isotopes (δ^13^C) vary substantially among primary producers and therefore mainly reflect primary carbon sources. The distribution of individuals in isotopic space is often used to quantify niche size^12^. Several aspects of trophic diversity and dietary divergence between individuals/species can be quantified and compared^13^. As we often lack the data to determine the true trophic niche of consumers in field studies, particularly for omnivores and deposit-feeders, use of the isotopic niche as a proxy is becoming common in ecological and evolutionary studies^14^. The isotope niche of a population integrates temporal (seasonal to multi-year) and – at least to some extent – spatial (population habitat) variations^14^. This quantitative approach to describe trophic space by stable isotopes has been used to test ecological theories^15^ including trophic ecology of invasive species in their novel environments^16^,^17^, but has also been criticized^18^. To evaluate how well the isotopic niche aligns with the trophic niche, more studies are needed that give the additional information required to infer ecological mechanisms from stable isotope data.

The Baltic Sea is a good model system for testing hypotheses on community structure and function as its low number of species makes it easier to elucidate trophic relationships^19^,^20^ and food-web effects of invasive species^8^,^21^. A mere three macrofaunal species constitute the native deposit-feeding community in sub-thermocline soft bottoms in the northern Baltic Sea. During the last two decades, three deposit-feeding species of the polychaete genus *Marenzelleria* spp. have invaded the Baltic Sea^22^, with mainly *Marenzelleria arctia* co-occurring with the native species below the summer thermocline at 15–20 m depth^23^. Today, *M. arctia* has become one of the most abundant species in many Baltic Sea areas covered by the Swedish National Monitoring Program (data available at www.smhi.se).

In temperate waters, deposit-feeders depend crucially on the sedimentation of the spring phytoplankton bloom as a source of food^21^,^24^,^25^. This is especially true for the amphipod *Monoporeia affinis* in the Baltic Sea, and its population decline over the last two decades has been linked to a reduced spring-bloom input^24^. A recent experimental study using isotope enriched algae found little overlap in use of freshly deposited spring bloom material between *M. arctia* and the native species, suggesting that *M. arctia* is not a strong competitor for the nutritious diatom input during the experimental duration of three weeks^21^. However, due to efficient utilization of old organic matter by *M. arctia*, competition can be expected to intensify once the spring bloom input to the sediment is exhausted and food limitation sets in^21^. The well-studied trophic ecology of the native species, especially the habitat and resource partitioning between the amphipods *M. affinis* and *Pontoporeia femorata*^20^,^26^,^27^ provides a solid background for applications of isotope niche in ecological studies.

Here, we compare the isotopic niches of the three native species and the non-indigenous *M.* *arctia* that together constitute the deposit-feeding community in a coastal area of the northern Baltic proper, where the invader has co-existed with the native species for about 20 years. To explain the invasion success of *M.* *arctia* in the Baltic Sea, we tested two alternative hypotheses:

1. There is little or no isotopic niche overlap between the co-existing species; this would indicate that *M. arctia* has occupied a vacant niche.
2. The invasive polychaete has a larger isotopic niche than the native species; this would suggest that *M. arctia* is a generalist feeder potentially outcompeting native species.

We also tested relationships between isotopic niche area and body condition to provide a better understanding of ecological implications of the observed variation in niche space and to test whether a large isotopic niche may confer growth benefits, as an expected outcome of generalist feeding^28^. Finally, C:N stoichiometry of the deposit-feeders and a relationship between sediment-consumer δ^15^N enrichment and food quality^29^, as measured by nitrogen content in sediment, were evaluated to understand differences in δ^15^N of the consumers.

## Results

### Species composition and sediment properties for each station and year

**Figure 1:**
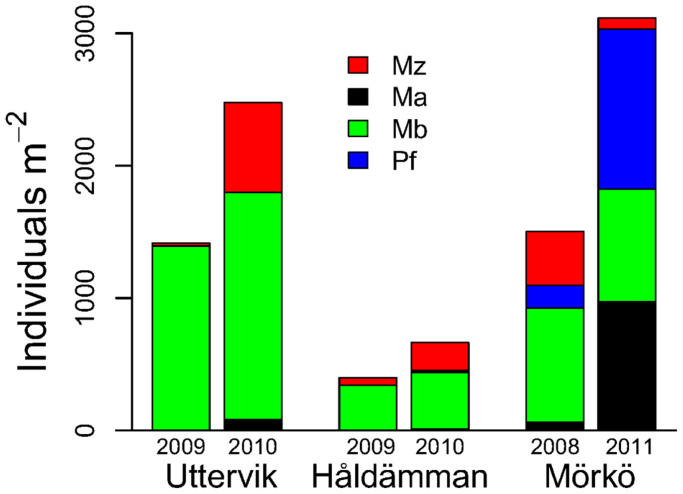
Species composition and abundance of benthic deposit-feeders at the sampling stations in 2009 and 2010 or 2008 and 2011. Ma = *Monoporeia affinis,* Mz = *Marenzelleria arctia*, Mb = *Macoma balthica* and Pf = *Pontoporeia femorata*. Samples at stn Mörkö were collected in October resulting in two year classes of amphipods being present, whereas only one year class was found at the other stations, which are sampled in May.

The total and relative abundance of species varied among stations, with densities of the invasive species *Marenzelleria arctia* ranging from 23 to 677 individuals m^−2^ (Fig. 1). As a group, deposit-feeders about doubled in abundance from 2009 to 2010 at all stations. Sediment carbon and nitrogen content were 5.0% and 0.7% (Håldämman), 5.2% and 0.7% (Uttervik) and 3.5% and 0.5% (Mörkö), respectively (n = 6–8, analytical precision within 0.1%).

### Isotopic niche overlaps

**Figure 2:**
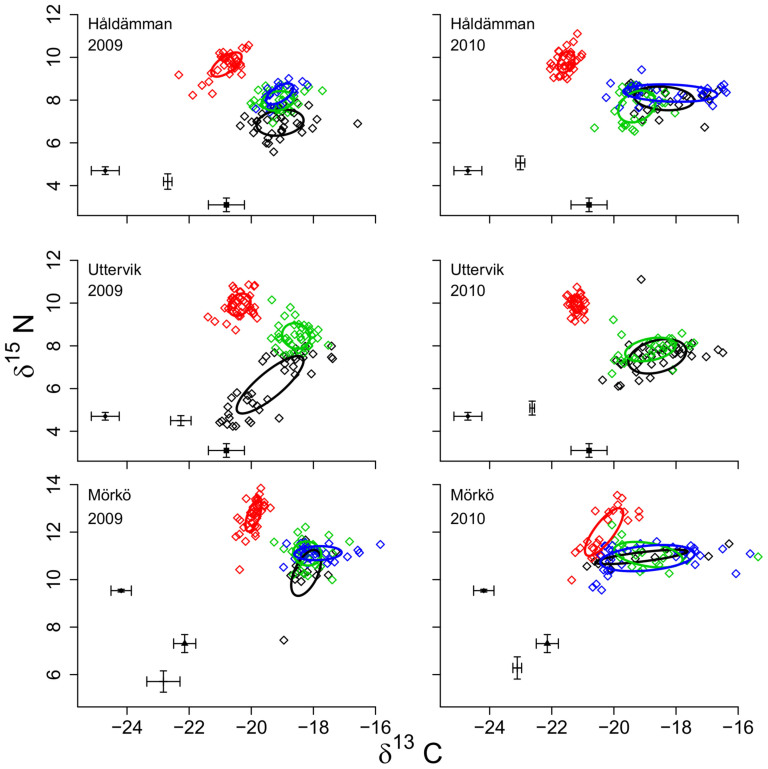
Stable isotope bi-plots illustrating the isotopic niche of the deposit-feeding community at the sampling stations. The invader *Marenzelleria arctia* (red) and the native co-occurring species, *Monoporeia affinis* (black), *Macoma balthica* (green), and *Pontoporeia femorata* (blue) at three stations in 2009 and 2010. All sampling events are pooled within years. The lines enclose the standard ellipse area (SEAc). Overlap between species is reported in Table 1. Sediment isotope value is denoted +, early spring bloom isotope value is denoted ♦ and late spring bloom isotope value is denoted ▪. Seston (stn Mörkö only) is denoted ▴. Resource data are mean ± standard deviation.

The isotopic niche, measured as the standard ellipse area (SEAc), of the invasive species *M. arctia* differed clearly from those of the native species in all datasets (Fig. 2). By contrast, SEAc values for native species overlapped on five of six occasions (Table 1). In 2010 at stn Mörkö, the niche area overlap among the three native species was nearly complete (Fig. 2), with *Monoporeia affinis* overlapping by 98% with *Pontoporeia femorata*. The highest median niche area overlap was found between *P. femorata (*58% of its niche area) and *Macoma balthica* (51%; n = 4).

### Species-specific differences in niche indices and relation to body condition

**Figure 3:**
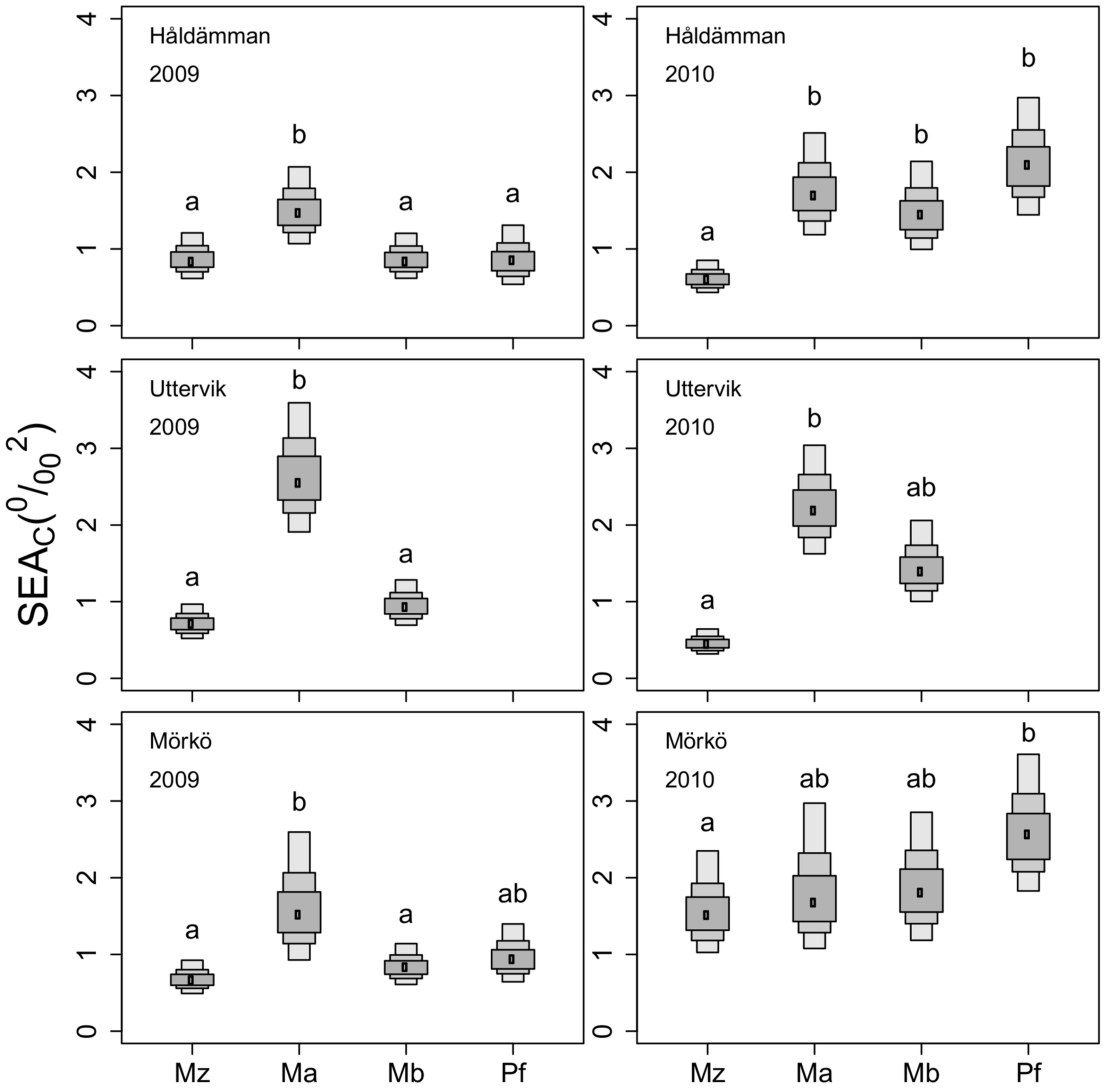
Mean standard ellipse area (SEAc) for each species at the sampling stations. Boxed areas indicates the SEA_B_ with Bayesian 50, 75 and 95% credible interval (Ma = *Monoporeia affinis,* Mz = *Marenzelleria arctia*, Mb = *Macoma balthica* and Pf = *Pontoporeia femorata*) at the three stations in 2009 and 2010. Shared common letters denote no significant difference (P > 0.05).

**Figure 4:**
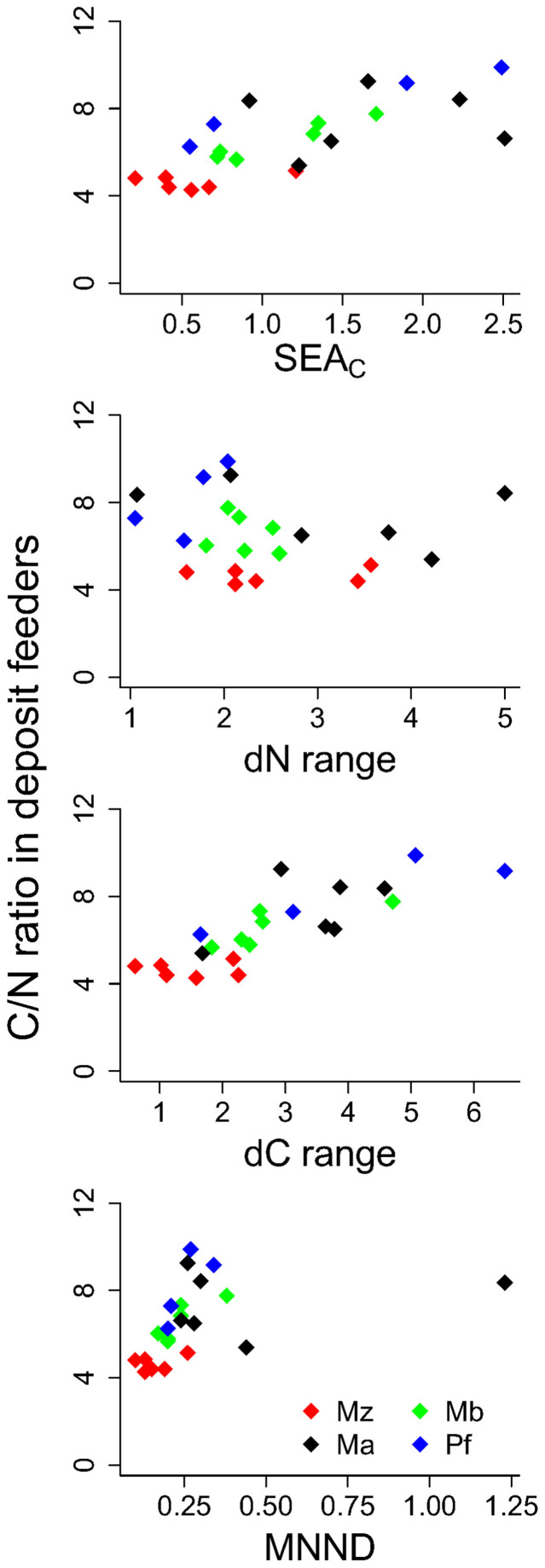
Body condition expressed as C:N ratio and isotope diversity indices. Top panel: Standard ellipse area, SEAc; mid panels: δ^15^N range and δ^13^C range and bottom panel: mean nearest-neighbour distance, MNND) for each species, station and year. Ma = *Monoporeia affinis,* Mz = *Marenzelleria arctia*, Mb = *Macoma balthica* and Pf = *Pontoporeia femorata*. Results from Pearson product moment correlations are reported in the text.

*M. affinis* or *P. femorata* had the largest and *M. arctia* the smallest SEA_B_ (SEA estimated via Bayesian interference) of all species (Fig. 3). A similar pattern was found for the other niche indices (Fig. 4). *M. arctia* had significantly smaller δ^13^C-range (indicative of the range of basal resources utilized) range than *M. affinis* and *P. femorata* (F_3,18_ = 4.75, p = 0.015) and smaller dietary divergence reflected in MNND (mean nearest neighbour distance) than *M. affinis* (F_3,18_ = 4.93, p = 0.011), but there was no difference in the δ^15^N-range between any of the species (F_3,18_ = 2.53, p = 0.089). Despite the low sample size, SEA_C_ of both *P. femorata* and *M. balthica* were significantly positively correlated to their C:N ratio, indicative of body condition (Pearson r = 0.98, p = 0.02, n = 4 and r = 0.96, p < 0.01, n = 6, respectively; Fig. 4). For *M. balthica*, C:N ratio and δ^13^C-range as well as MNND were also positively correlated (Pearson r = 0.80, p = 0.05 and r = 0.84, p = 0.43, respectively, Fig. 4), but not the C:N and δ^15^N-range (p > 0.6, Fig. 4). For *M. affinis* and *M. arctia* there were no significant correlations between SEAc or any of the other niche diversity indices and the C:N ratio (p > 0.3 in all cases, Fig. 4). Also, in no case was a significant correlation between SEAc of native species and *M. arctia* density observed (Pearson r < 0.1, p > 0.1).

### Sediment-consumer enrichment in δ^15^N and nitrogen content in sediment

**Figure 5:**
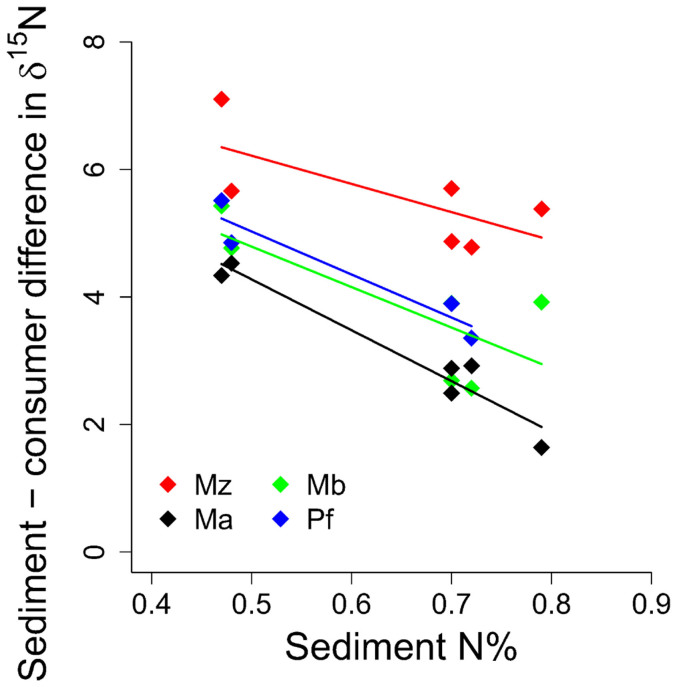
Negative relationship between consumer enrichment in relation to the sediment δ^15^N values and nitrogen content in sediment. Average values for station and year. Ma = *Monoporeia affinis,* Mz = *Marenzelleria arctia*, Mb = *Macoma balthica* and Pf = *Pontoporeia femorata*. Results from GLM is reported in Table 2.

There was a significant decrease in the enrichment values in consumers with increasing %N in sediment. Moreover, *M. arctia*, which had the lowest C:N ratio among the species tested (Fig. 4), also had significantly higher δ^15^N values than the three native species, which may reflect greater limitation by low protein quality in sediments (Table 2, Fig. 5).

## Discussion

The isotopic niche concept is increasingly used by ecologists but there are still too few studies that evaluate how well the isotopic niche aligns with the trophic niche. The well-studied trophic ecology of our deposit-feeding community allows us to discuss whether the assumptions underlying isotopic niche interpretations may have been violated in this system, with implications for the interpretation of diets and trophic positions in higher level consumers of general interest in ecology.

The isotopic niche of the non-native *Marenzelleria* *arctia* was clearly distinct from those of all native species in the deposit-feeding guild, thus supporting Hypothesis 1. The clear separation between *M. arctia* and the native species, regardless of *M. arctia* population density and organic content of the sediment, suggests limited competition for resources and provides evidence that this invasive species has occupied a vacant niche in the Baltic Sea soft-bottom habitat. The field data presented here strongly support experimental findings showing efficient resource partitioning between *M. arctia* and the native species^21^. The species poverty of Baltic sediments, common to other brackish environments where osmoregulation capacity limits species distribution^30^, has likely left more niches vacant than in more species-rich systems. Such vacant niches can – at least in part – explain the high invasibility of brackish waters^31^. The consequences of invasion-induced species richness for ecosystem productivity are complex even in a species-poor system like the Baltic Sea^21^, but the well-known trophic ecology of the studied macrofaunal species gives insight into the mechanisms behind the observed patterns. Below we discuss them in detail.

The isotopic niche of *M. arctia* differs from those of the native species in three main ways: more enriched δ^15^N, more depleted δ^13^C and, compared to the amphipods, a smaller niche area as well as lower values for the other niche indices representing basal resources and dietary divergence between individuals. The relatively narrow isotopic niche of *M. arctia* and the absence of a positive relationship between its body condition and niche size suggests specialized feeding, in contrast to our Hypothesis 2. It is, thus, likely that *M. arctia* occupies a previously vacant niche. The resemblance of its carbon isotope value to both the sediment and diatom carbon isotope would indicate a strong dependence on the diatom input. *M. arctia* do feed on diatoms during experimental conditions but grow nearly as well when offered aged sediment only^21^, whereas native species are known to critically depend on the diatoms for growth and survival^21^,^24^,^25^. After lipid correction^32^, the carbon isotope signature of the native species became however clearly heavier than both the sediment and diatom signatures (Fig. 2), which is most likely related to amphipod' higher requirements for carbon to build lipid reserves^20^,^21^,^33^ and, therefore, less discrimination against the heavier isotope. Furthermore, the dissolved inorganic carbon pool in the water becomes more enriched as the season progresses^34^, resulting in more enriched carbon values in phytoplankton later in the season, as evident from the late spring bloom isotope data that better resemble the carbon signal of the native species (Fig. 2). Although dinoflagellates, which dominate the late spring bloom, are a carbon rich food source, they settle mostly as cysts which are not efficiently digested by macrofauna^35^, and their value as food for benthos may therefore be questioned. It is, however, important to remember that the phytoplankton or seston isotope signatures do not necessarily reflect the isotope signal of decomposing organic material which has undergone a microbial conditioning in the sediment that may enrich isotope values^36^. A high contribution of microbially processed organic matter might explain the higher δ^15^N of *M. arctia.*

An alternative explanation for greater δ^15^N enrichment of *M. arctia* could be a higher isotopic fractionation by the polychaete, due to a mismatch between the nitrogen requirements and its availability in the sediments. It has been hypothesized that nitrogen isotopic discrimination should decrease as dietary protein quality increases^29^, and the significant negative relationship between sediment-consumer enrichment in δ^15^N and sediment %N observed in our study (Fig. 5) supports this hypothesis. Moreover, as *M. arctia* has relatively high nitrogen requirements^21^ due to its nitrogen content of about 10% compared to < 8% for the native species^37^, the higher enrichment observed for the polychaete (Fig. 5) is not surprising. Indeed, a higher flux of amino acids through various metabolic pathways, where transamination and deamination take place, should result in elevated^15^N fractionation, particularly when food is suboptimal in terms of the protein quality and similarity in the essential amino acid composition^38^. Finally, the high δ^15^N signal could indicate that *M. arctia* has a higher trophic position by feeding also on meiofauna or on dead macrofauna. This, however, seems unlikely as *Marenzelleria* spp. are classified as facultative suspension- (surface) deposit-feeders^39^ and have been shown to efficiently incorporate labelled phytodetritus^21^. Furthermore, Viitasalo^40^ and Urban-Malinga et al.^41^ found no evidence of predation by *Marenzelleria* spp. on cladoceran resting eggs or meiofauna in experimental studies. We therefore suggest that isotope enrichment originating from recycling of N in the benthic food web and greater organismal fractionation by the polychaete are the main causes of higher δ^15^N in *M. arctia* than in the native species. We note that assigning the high δ^15^N to predatory feeding behaviour in this species in the analysis of the benthic food web structure would lead to erroneous interpretations of the trophic positions of higher level consumers, such as benthivorous fish, when this signal is translated higher up in the food web.

Isotopic niche assessment relies on several assumptions, namely: (i) there is no isotopic overlap in food sources; (ii) isotopically identical food sources should yield identical delta values, i.e. variability in diet-consumer fractionation is negligible; (iii) temporal integration is similar between the species, i.e. the isotopic signatures reflect the diets assimilated over similar periods; and (iv) all species are in isotopic equilibrium with their diets. Hence, it is important to critically evaluate how well the isotopic niche aligns with the trophic niche. Assumption (i) is violated since both diatoms and sediment have very similar isotope values, although none can be directly considered equivalent of the isotope signal of the assimilated diet. As discussed above, due to differential elemental requirements and biological stoichiometry, species- or stage-specific fractionation of nitrogen and carbon isotopes would likely violate assumption (ii)*.* Indeed, variation in growth and metabolic rates (e.g., *M. affinis* has faster growth, respiration rate and lipid accumulation than *P. femorata*^33^) is likely to result in differential fractionation^42^, violating assumptions (ii), but also (iii) and (iv) and, hence, affecting the interpretation of isotopic niche indices and niche overlap for these species. Differences in growth rate between long-lived bivalves and semelparous amphipods should then be even more pronounced, with similar implications as above. Moreover, asynchronous moulting in crustaceans as a function of individual growth rate would further increase δ^15^N variability among individuals in a mixed population^43^ compared to a population with strong cohort structure. Therefore, isotopic niche area comparisons should be interpreted with caution and high isotopic niche overlap observed between the native species (up to 100%), should not be considered as a proof of equally extensive trophic overlap.

Indeed, the amphipod species with largely overlapping trophic niches partition resources by depth in sediment when found together, with *P. femorata* feeding deeper in the sediment^20^,^21^,^26^,^27^. Such context-dependent small-scale partitioning is not likely to be reflected in isotopic niche overlap, hence violating assumption (i). In contrast, the overlap between *M. affinis* and *M. balthica* isotopic niches likely reflects at least some food competition, since both species are known to feed in the top centimetre of the sediment^21^. Moreover, asymmetrical competition for phytodetritus between these species was suggested in the latter study, with incorporation rates in *M. affinis* negatively influenced by the presence of *M. balthica.* This, and predation by *M. affinis* on larval *M. balthica*^44^, could explain why these species seldom co-occur in high abundances^45^. Increased competition for resources could result in trophic niche widening, if the consumers are forced to broaden their diets and shift to suboptimal resources in order to meet energy requirements^46^. However, the common view is that it is a lack of competition rather than increased competition that allows consumers to extend their realised niche^47^. In support of this, we found a positive correlation between isotopic niche size and body condition (reflected by C:N ratio) for *P. femorata* and *M. balthica*, suggesting that supplemental feeding is physiologically advantageous^28^.

Studies of invasive species often examine their deleterious effects on native species, community composition, food-web functioning and ecosystem services. Previous research focused on competitive interactions between invasive *Marenzelleria* spp. and native deposit-feeders^48^,^49^ since its invasion in the northern Baltic Sea coincided with population crash in *M. affinis*. Recent evidence indicates, however, that the observed crash in amphipod abundance was an effect of climate-induced food shortage, rather than the invasion^24^. Moreover, it was later found that there are three species of the genus *Marenzelleria* in the Baltic, which differ in habitat requirements^23^ and sediment reworking^50^, making it difficult to interpret some of the early interspecific competition experiments^48^,^49^ and to extrapolate results from shallow^51^ to deeper waters. Furthermore, the capacity of *Marenzelleria* spp. to bury deeper in the sediment than the native species is feared to facilitate remobilization of old contaminants to the overlying water^52^. On the other hand, several recent studies have suggested positive effects of the *M.* cf. *arctia* invasion in deeper areas of the Baltic Sea, including enhanced long-term retention of phosphorus in sediments through increased oxygenation^53^, burial of fresh detritus deeper in the sediment where mineralization is lower^21^,^54^ and suppressed cyanobacterial recruitment^55^,^56^, counteracting eutrophication symptoms. Finally, as discussed above, the results of our field study and the earlier experiment with *M. arctia*^21^ indicate that this invader and the native species show resource partitioning that may enhance resource usage and energy transfer efficiency through broadening the community's trophic niche. That *M. arctia* can grow rapidly on sediment lacking recent bloom input^21^ means that detritus that would otherwise have been lost to bacterial mineralization is now efficiently converted to secondary production and ultimately to fish production*.* It is not known to what extent *M. arctia* can replace declining *M. affinis* populations as fish food, but *Marenzelleria* spp. is eaten by sand gobies, which spend winter in deep water^57^, indicating that it can function as food for other benthivorous fish as well.

In conclusion, there is no isotopic niche overlap between *M. arctia* and the native deposit-feeding community during the growth season. This strongly indicates resource partitioning between the invader and the native species. The native species, on the other hand, often exhibit almost completely overlapping isotopic niches, although it is important to remember that this does not rule out resource partitioning in space (e.g., by sediment depth), variability in fractionation, or use of different foods with similar isotopic values, resulting in similar isotopic niches. Hence, caution in interpretation of the isotopic niche is necessary as it may not fully represent the trophic niche, and studies testing correspondence between trophic and isotopic niches in ecologically relevant settings are warranted. Although the wider food-web consequences of the *M. arctia* invasion are difficult to assess, as a food web component, *M. arctia* has likely enhanced resource utilization in the Baltic Sea, with potentially positive effects further up the food chain.

## Methods

### Sampling procedures and isotope analyses

We sampled sediment and deposit-feeding macrofauna during May to September in 2009 (4 sampling events) and 2010 (2–3 sampling events) at three coastal stations in the north-western Baltic proper; stn Håldämman (30 m depth, 58°49′ N, 17°34′ E), stn Uttervik (20 m depth, 58°50′ N, 17°32′ E), and stn Mörkö (23 m depth, 58°54′ N, 17°42′ E). We used a benthic sled, set to collect the top 1–2 cm sediment, which was then sieved through a 1 mm sieve to retain fauna. The two most abundant species, the bivalve *Macoma balthica* and the non-indigenous polychaete *Marenzelleria arctia*^23^ were found in high numbers at all stations on all sampling occasions. Benthic community composition data for May 2009 and 2010 were obtained from Swedish National Monitoring Programme (SNMP, data available at www.smhi.se; stations 6001 and 6010 near stn Uttervik and stn Håldämman, respectively). Benthic community data from stn Mörkö in October in 2008 and 2011 were obtained from the SYVAB's marine monitoring program in Himmerfjärden Bay (Himmerfjärden Eutrophication Study; www2.ecology.su.se. At stn Mörkö, the amphipod *Monoporeia affinis* was less abundant in May and absent in July 2009. The amphipod *Pontoporeia femorata* was never found at stn Uttervik and absent from stn Håldämman in May and July 2009 (Fig. 1). On each sampling occasion, we selected up to 10 individuals of similar size (2 mg dry weight, shell-free for *M. balthica*) of each species from each station for isotope analysis. In total, 655 individuals were analysed, about 200 each for *M. affinis, M. balthica* and *M. arctia* and 81 for *P. femorata.* Animals and bulk sediment (upper 1–2 cm, collected by the sled on each sampling occasion were oven-dried (60°C), packed individually into tin capsules and analysed for elemental and stable isotope content (carbon and nitrogen) at the UC Davis Stable Isotope Facility, USA. The C and N isotope ratios are expressed in the δ notation, using equation (1):
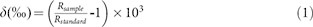
where R is the ratio between the heavy and light isotopes (^13^C: ^12^C or ^15^N: ^14^N). The stable isotope ratio, δ, is defined as the deviation from an international reference standard (Vienna PeeDee Belemnite for C, and atmospheric nitrogen gas for N), given in ‰ since it is a small figure. Higher δ indicates a higher proportion of the heavy isotope. Samples were run in continuous flow with a standard deviation of <0.2‰ among replicate standard samples both for C and N. Before calculations and statistical analyses, all δ^13^C-values were corrected for lipid content using the C:N ratio^32^.

Data on spring bloom isotopic signal originate from different sources (Table 3). Stable isotope data for early (dominated by the diatom *Thalassiosira* sp., n = 5) and late (dominated by *Dinophysis* sp. n = 5) spring bloom (March and April, respectively) for stations Uttervik and Håldämman were obtained from Rolff^58^. Spring bloom material from stn Mörkö was sampled in April (n = 3, sample dominated by *Thalassiosira* sp. but also containing ciliates and rotifers). Seston from mid-May was sampled at 20 m depth using water bottle sampler (22.7 L). The entire volume was filtered with 90 µm sieve and particular matter of the filtrate was collected on GFF (n = 3). All samples were oven-dried (60°C), packed into tin capsules and analysed for elemental and stable isotope content as described above.

### Data analyses and statistics

Isotopic diversity indices^13^ were calculated for each species, station and year using the SIBER (Stable Isotope Bayesian Ellipses in R) package for R v.2.10.1^59^. The isotope niche of a population integrates temporal (seasonal to multi-year) and – at least to some extent – spatial (population habitat) variations. To focus on our hypotheses, we pooled population data within a season as advocated by Antonio and Richoux^14^. Moreover, deposit- and suspension-feeders are commonly used in food web analysis to represent the baseline signal smoothing-out fluctuations in stable isotope signatures of primary producers that would obscure trophic position estimates in a food web^60^. Therefore, pooling population data within a season would incorporate variations related to both source signature and individual feeding preferences consistent with trophic niche definition.

Three of the indices developed by Layman et al.^13^ were used here; trophic diversity (dN, δ^15^N-range), multiple basal resources and a potential for niche diversification (dC, δ^13^C-range) and dietary divergence between individuals (mean nearest neighbour distance; MNND), which describes how individuals are distributed relative to one another within a population's dietary niche space. Instead of the convex hull area^13^, which is the total area encompassed by all points on a δ^13^C−δ^15^N bi-plot, we calculated the standard ellipse area, SEA^59^ (comparable to SD in univariate cases). SEA_C_ (c denotes that SEA was corrected for small sample size) provides information about the core aspects of a population's niche and is less sensitive to outliers and small samples sizes (n < 30 according to Syväranta et al.^61^). Overlap in SEA_C_ between species was calculated for each species combination. Differences in SEA_C_ between species were estimated via Bayesian interference (SEA_B_), according to Jackson et al.^59^. Differences in the other diversity indices (dN, dC and MNND) between species were tested using one-way ANOVA for each index separately, followed by Tukey's HSD. Residuals were inspected for normality and MNND data were log-transformed to improve distribution of the residuals. We also performed Pearson product moment correlations to explore possible relationships between (i) *Marenzelleria arctia* density and niche size (SEA_C_) of native species and (ii) SEA_C_ and the C:N ratio (high values indicate better body condition with larger lipid reserves) for each species. The effect of %N in sediment on sediment-consumer enrichment in δ^15^N was tested with general linear model (GLM) with sediment %N and C:N ratio of deposit-feeders as independent variables. ANOVA, GLM and Pearson correlation tests were performed in STATISTICA 12 (StatSoft Inc.). Raw data on isotope composition and C/N ratio are available from the Dryad Digital Repository: http://datadryad.org.

## Author Contributions

A.M.L.K. and R.E. designed the study, A.M.L.K. performed the field experiment, and A.M.L.K. and E.G. performed the statistical analyses. A.M.L.K. wrote the first draft of the manuscript and all authors contributed substantially to revisions.

## Additional information

**How to cite this article**: Karlson, A.M.L., Gorokhova, E. & Elmgren, R. Do deposit-feeders compete? Isotopic niche analysis of an invasion in a species-poor system. *Sci. Rep.* 5, 9715; DOI:10.1038/srep09715 (2015).

## Figures and Tables

**Table 1:** Percentage of isotopic niche area overlap by species (underlined) for every species combination at each station and year (Ma = *M. affinis*, Mb = *M. balthica* and Pf = *P. femorata*). NA denotes a non-existing species combination. *M. arctia* is not included because it never overlapped with the native species. Values in brackets (stations Håldämman 2009 and Mörkö 2009) are for late summer only; *P. femorata* and *M. affinis* were absent or only found in low numbers in early summer at these stations

Species combination	Håldämman 2009	Håldämman 2010	Uttervik 2009	Uttervik 2010	Mörkö 2009	Mörkö 2010
**Ma-Mb**	0 (0)	25	0	46	26 (29)	67
**Ma-Mb**	0 (0)	32	0	78	44 (35)	36
**Ma-Pf**	0 (0)	62	NA	NA	16 (9)	98
**Ma-Pf**	0 (0)	54	NA	NA	27 (33)	36
**Mb-Pf**	46 (49)	19	NA	NA	55 (33)	85
**Mb-Pf**	62 (80)	14	NA	NA	57 (100)	58

**Table 2:** GLM testing effects of nitrogen content in sediment (%N) and C:N ratio of consumers on the sediment-consumer enrichment in δ^15^N (see Fig. 5)

Effect	Estimate	SE	Wald stat	P-value
Intercept	10.28	0.94	119.32	<0.001
C:N ratio	−0.47	0.10	22.51	<0.001
Sediment N%	−4.63	1.34	11.93	<0.001

**Table 3:** Resource data for deposit-feeders at each station (H = Håldämman, U = Uttervik and M = Mörkö). See methods section for details on spring bloom species composition and Fig. 2 for isotopic signature of each resource in relation to consumer signatures

Resource	Year	Month	Station	Sample preparation	Storage conditions
**Spring bloom**	1994*	March-April	H, U	0-10 m depth, 50-100 μm sieve	Frozen, oven dried
**Spring bloom**	2008	April	M	0-10 m depth,100 μm sieve	Frozen, oven dried
**Seston**	2008	May	M	20 m depth, GFF	Oven dried
**Sediment**	2009- 2010	May-Sept.	H, M,U	Benthic sled, 500 μm sieve	Oven dried

*Rolff^59^.
